# Cisplatin inhibits MEK1/2

**DOI:** 10.18632/oncotarget.4355

**Published:** 2015-06-20

**Authors:** Tetsu Yamamoto, Igor F. Tsigelny, Andreas W. Götz, Stephen B. Howell

**Affiliations:** ^1^ Moores Cancer Center and Department of Medicine, University of California, San Diego, La Jolla, CA 92093; ^2^ Neuroscience Department, University of California, San Diego, La Jolla, CA 92093; ^3^ San Diego Supercomputer Center, University of California, San Diego, La Jolla, CA 92093

**Keywords:** MEK1, RAS, ERK, copper, cisplatin

## Abstract

Cisplatin (cDDP) is known to bind to the CXXC motif of proteins containing a ferrodoxin-like fold but little is known about its ability to interact with other Cu-binding proteins. MEK1/2 has recently been identified as a Cu-dependent enzyme that does not contain a CXXC motif. We found that cDDP bound to and inhibited the activity of recombinant MEK1 with an IC_50_ of 0.28 μM and MEK1/2 in whole cells with an IC_50_ of 37.4 μM. The inhibition of MEK1/2 was relieved by both Cu^+1^ and Cu^+2^ in a concentration-dependent manner. cDDP did not inhibit the upstream pathways responsible for activating MEK1/2, and did not cause an acute depletion of cellular Cu that could account for the reduction in MEK1/2 activity. cDDP was found to bind MEK1/2 in whole cells and the extent of binding was augmented by supplementary Cu and reduced by Cu chelation. Molecular modeling predicts 3 Cu and cDDP binding sites and quantum chemistry calculations indicate that cDDP would be expected to displace Cu from each of these sites. We conclude that, at clinically relevant concentrations, cDDP binds to and inhibits MEK1/2 and that both the binding and inhibitory activity are related to its interaction with Cu bound to MEK1/2. This may provide the basis for useful interactions of cDDP with other drugs that inhibit MAPK pathway signaling.

## INTRODUCTION

Cisplatin (cDDP) is widely used for the treatment of many types of cancer. It is a polar molecule that does not readily diffuse across bilayer lipid membranes as shown by the fact that it remains stably encapsulated in liposomes [1]. Recent studies have demonstrated that cDDP influx and efflux are modulated by transporters and chaperones involved in copper (Cu) homeostasis in mammalian cells [reviewed in [2]]. Cu is an essential micronutrient, but because it can readily undergo redox reactions under intracellular conditions it can generate reactive oxygen species that can be dangerous to cells. This threat is managed in mammalian cells by a group of Cu-binding chaperones and efflux transporters that bind Cu in CXXC motifs and keep its free concentration to <10^−18^ M [3]. Cu entering on the major influx transporter CTR1 is handed to one of the Cu chaperones ATOX1, CCS or COX17 and the affinity of the CXXC motifs in these proteins is such that they can subsequently donate the Cu to the CXXC motifs in the Cu efflux transporters ATP7A and ATP7B, or to the various Cu-dependent enzymes [4].

The discovery that the major Cu influx transporter CTR1 modulates the uptake of cDDP [reviewed in [2]] introduced the concept that cDDP exploits chemical characteristics that make it similar to Cu^+1^ to get into the cell [5]. Both Cu and cDDP are soft Lewis acids and share the electronic characteristics that allow weak binding to methionines and to CXXC motifs. The ability of cDDP to kill cells despite the great excess of GSH in the cytoplasm, with which it readily reacts, led us to speculate that, as it does for Cu, the Cu homeostasis system operates to protect cDDP as well as to distribute it to specific sites within the cell [5]. This concept depends on the idea that cDDP, like Cu, can transiently associate with Cu binding proteins that protect it against reaction with thiols, and that it can undergo transfer from one such protein to another. Our studies and those of others have now established that some of these steps are feasible in extracellular systems. The CXXC motifs in ATOX1 [6–8], and ATP7B [9] bind cDDP, and ATOX1 can transfer it to the CXXC motif in the second MBD of ATP7B [8, 10]. It has also been established that a complex of methionine with cDDP can transfer the cDDP to the N7 of guanine in DNA [11]. Recent findings indicate that CTR1 is important to receptor tyrosine kinase signaling [12–14] and there is evidence that the Cu delivered by CTR1 is required for SOD1 to generate the H_2_O_2_ that limits receptor phosphatases [14] and for the activity of MEK1 which turns out to be a Cu-dependent enzyme [13, 15].

The ability of cDDP to bind to the CXXC motifs of the Cu homeostasis proteins raises the question of whether cDDP can also bind to other types of Cu binding sites found in Cu-dependent enzymes, or whether it can interfere with their activity by limiting the transfer of exchangeable Cu onto the enzymes. Given the importance of MEK1/2 in the MAPK pathway, and the recent discovery that MEK1/2 is a Cu-dependent enzyme, we examined the ability of cDDP to bind and to inhibit MEK1 in both a cell-free system and in whole cells. We report here that cDDP binds to and inhibits MEK1 in both settings, and that inhibition is offset by increasing the availability of Cu.

## RESULTS

### cDDP inhibits the activity of recombinant MEK1 *in vitro*

**Figure 1:**
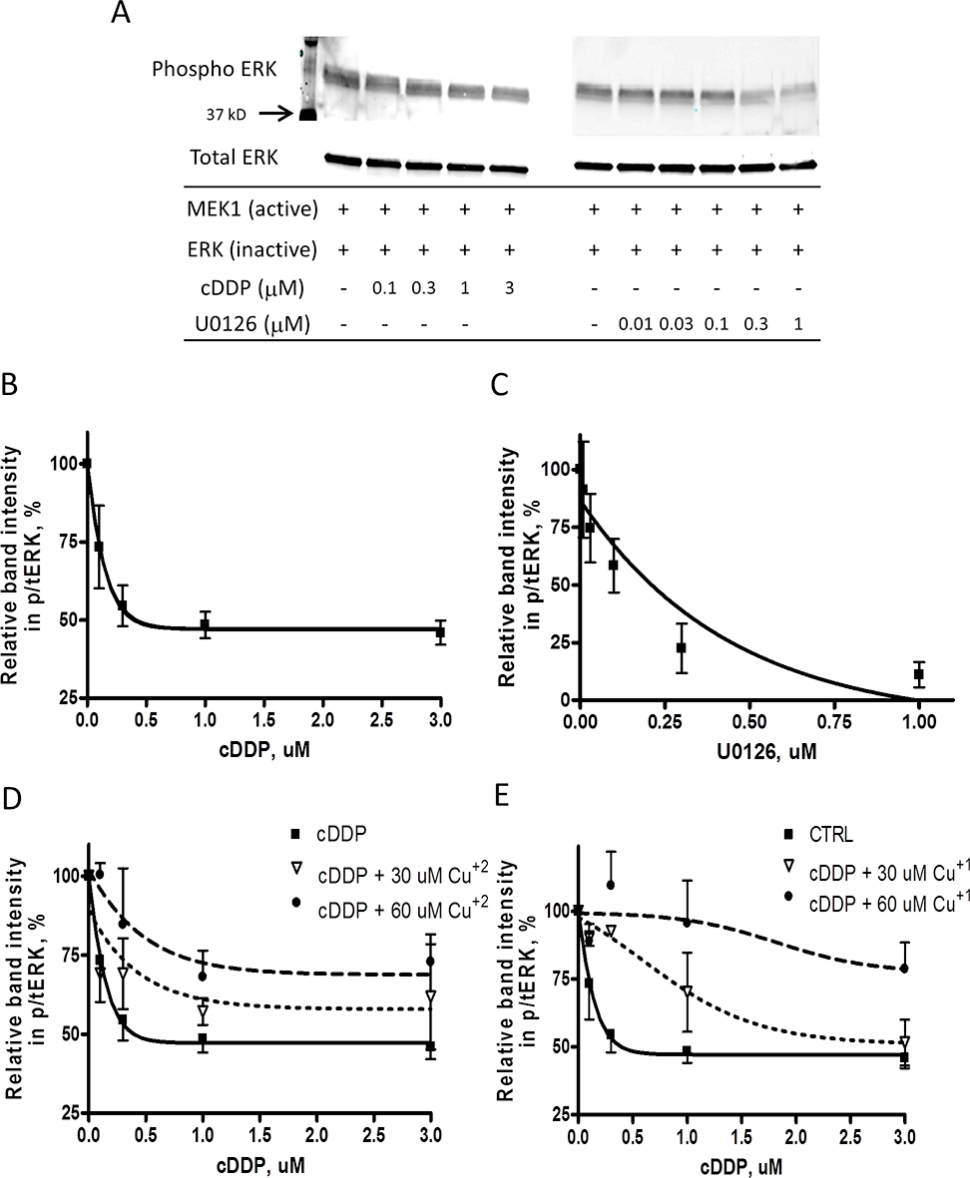
Inhibition of the activity of recombinant MEK1 by cDDP and U0126 **A.** Representative Western showing inhibition of MEK1 by cDDP and U016. **B. and C.** MEK1 activity as a function of cDDP and U0216 concentration in the absence of Cu. **D. and E.** Effect of increasing concentrations of Cu^+2^ and Cu^+1^ on inhibition of MEK1 by cDDP. Mean data from 3–5 experiments was fitted by PRISM to produce the curves. Data are presented as mean ± SEM.

MEK1 is an important enzyme in the MAPK pathway and has recently been identified as a Cu binding protein although the details of how it binds Cu are unknown. The effect of Cu and cDDP on the activity of recombinant MEK was tested by assaying the ability of an active form of MEK1 to phosphorylate ERK1/2 in the presence of increasing concentrations of the two compounds. The non-competitive MEK inhibitor U0126 was used as a positive control, and the formation of pERK was monitored by Western blot analysis. As shown in Figure 1A–1C both cDDP and U0126 inhibited the activity of recombinant MEK1. The IC_50_ for cDDP, calculated as the concentration that reduced activity by half of the maximum inhibition attainable, was 0.28 ± 0.09 μM whereas for U0126 was 0.21 ± 0.11 μM. Figure 1D shows that addition of CuSO_4_, which is largely Cu^+2^, reduced the maximal inhibition attainable by cDDP in the dose dependent manner, and Figure 1E shows that increasing concentration of CuSO_4_ in the presence of dithiotheitol (DTT) that keeps the Cu reduced to Cu^+1^ even more potently shifted the cDDP inhibition curve to the right suggesting that both forms of Cu modulate the interaction of cDDP with MEK1.

### cDDP binds to recombinant MEK1

**Figure 2:**
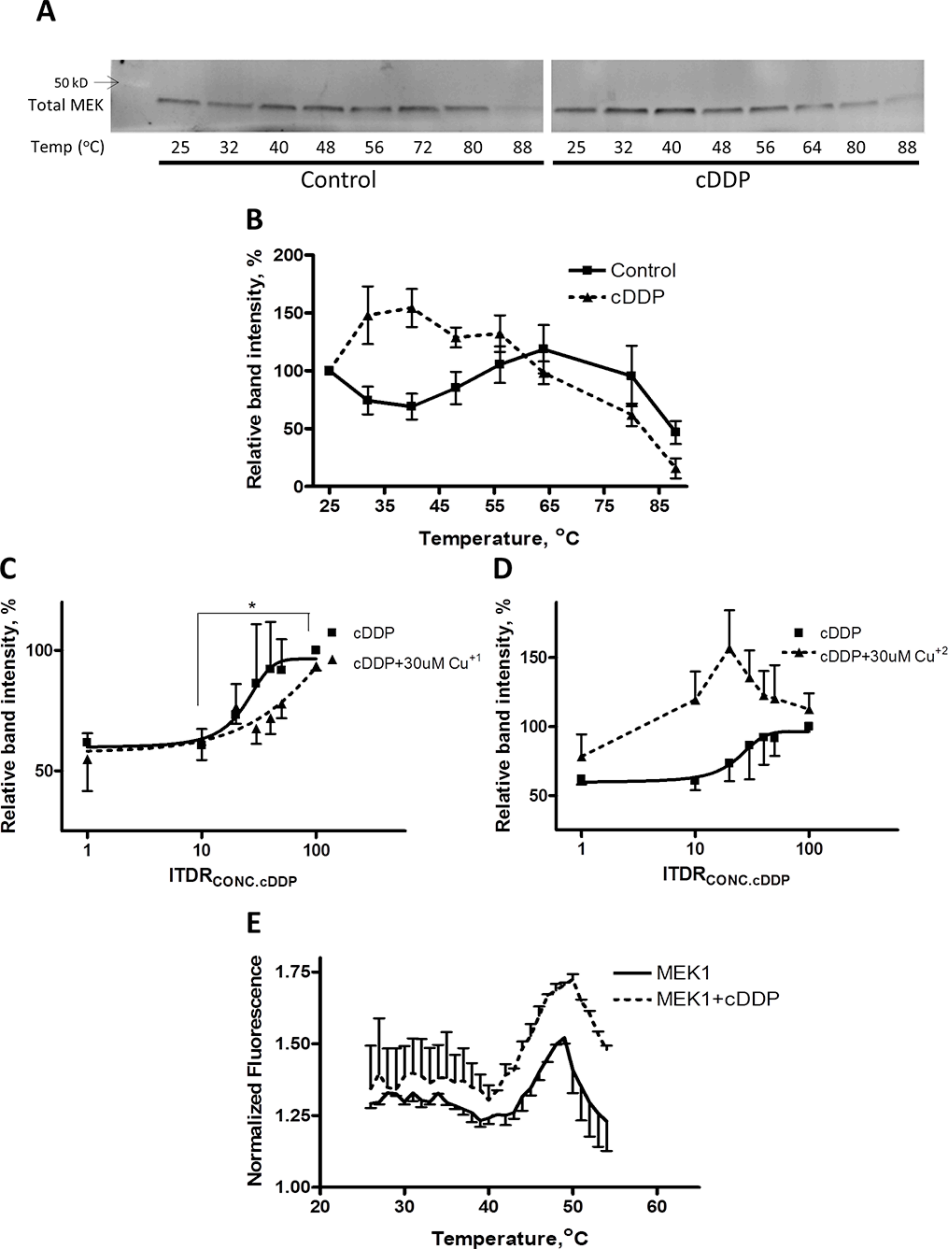
Thermal stabilization of recombinant MEK1 by cDDP **A.** Representative Western blot showing stabilization of MEK1 by 30 μM cDDP. **B.** MEK1 level as a function of temperature in the absence and presence of 30 μM cDDP. **C. and D.** ITDR curves for stabilization of MEK by cDDP at 45°C in the absence or presence of 30 μM Cu^+1^ or Cu^+2^ (*p* < 0.05 for the difference between baseline and maximal effect of cDDP). **E.** Melting curve of MEK1 in the absence and presence of 30 μM cDDP as determined by differential scanning fluorimetry. Data are presented as mean ± SEM, *N* = 3.

**Figure 3:**
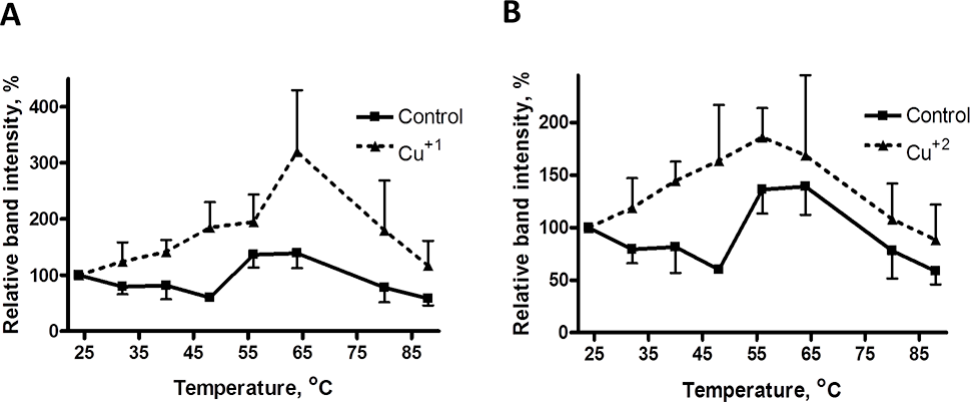
Thermal stabilization of recombinant MEK1 by Cu MEK1 level as a function of temperature in the absence and presence of 30 μM Cu^+1^ (panel **A**) and Cu^+2^ (panel **B**). Data are presented as mean ± SEM, *N* = 3.

The ability of Cu to offset the inhibitory effect of cDDP on MEK1 raises the question of whether the interaction of cDDP with MEK1 interferes with the binding of Cu to MEK1 or the ability of Cu to optimally configure the structure of the enzyme. To explore this we tested the ability of cDDP to prevent the thermal denaturation of recombinant MEK1. We studied the effect of Cu as well to confirm that MEK1 is a Cu binding protein and determine whether they both produce the same pattern of stabilization. As shown in Figure 2A and 2B, MEK1 had an unusual thermal denaturation curve with loss of stability at temperatures up to 45°C but some recovery of stability between 45 and 65°C. The shape of this curve was similar in each of the 3–5 independent experiments performed (see also Figure 3A and 3B). A concentration of 50 μM cDDP increased the stability of MEK1 at the lower temperatures but had little effect at the higher temperatures (Figure 2B). Figure 2C shows the isothermal dose-response fingerprint (ITDRF) for cDDP in the absence and presence of Cu^+1^ that indicates that the stability of recombinant MEK1 increases as a function of cDDP concentration and that Cu^+1^ shifts the curve to the right suggesting an element of antagonism. On the contrary, Cu^+2^ shifts the curve to the left indicating that Cu^+2^ increases the effect of cDDP on recombinant MEK1 stabilization (Figure 2D). This result suggests a complex interaction between cDDP and Cu with respect to thermal stability that depends on the oxidation state of the Cu.

To confirm that cDDP interacts directly with MEK1 a second assay was used in which the ability of the dye SYPRO orange to bind to hydrophobic portions of MEK1 that become exposed as the temperature is increased was determined in the presence and absence of cDDP. The data presented in Figure 2E indicate that cDDP did not alter the MEK1 melting temperature (Tm) of 45°C but did increase SYPRO orange binding at all temperatures. This result is consistent with the conclusion that cDDP interacts directly with MEK1 to cause exposure of hydrophobic portions of MEK1 irrespective of temperature. This effect was not due to an interaction between SYPRO orange and cDDP (data not shown).

We also examined the effect of both Cu^+1^, as CuSO_4_ in the presence of DTT (Figure 3A) and Cu^+2^, as CuSO_4_ in the absence of DTT (Figure 3B) on the thermal stability of MEK1. Both forms of Cu stabilized MEK1 although the peak effect of Cu^+1^ was higher than that of Cu^+2^. These data indicate that both reduced and oxidized forms of Cu can stabilize recombinant MEK1 against thermal denaturation.

### cDDP inhibits MEK1/2 in whole cells

To determine whether cDDP inhibits the MAPK pathway in whole cells under basal and oncogene driven conditions we used 10T1/2 mouse fibroblasts that had been molecularly engineered to express the avian tumor virus receptor A so that they could be infected with avian retroviruses expressing different oncogenes. For this study we used the parental cells infected with an empty vector (CTRL) and a subline that had been infected with a vector expressing an oncogenic human H-Ras mutant so as to be able to examine the effect of cDDP on untransformed cells with just basal MAPK activity, and in isogenic cells with increased MAPK activity that drives transformation. The ability of each cell line to form colonies in soft agar was tested to confirm the transformed phenotype. Supplementary Figure 1A shows that, after an incubation of 14 days, while the empty vector- infected CTRL cells formed few colonies, the H-Ras oncogene-expressing cell line formed a large number of colonies indicating its acquisition of the cell-autonomous growth characteristics of transformed cells. To determine the effect of oncogenic H-Ras expression on sensitivity to cDDP, the CTRL and H-Ras-expressing cells were exposed to increasing concentrations of cDDP for 72 h after which viability was determined using the CCK-8 assay. The concentration-survival curves were completely superimposable indicating no difference in cDDP sensitivity (Supplementary Figure 1D). The IC_50_ for the CTRL cells was 37.4 ± 2.7 and for the H-Ras expressing cells it was 39.3 ± 6.7 μM.

The extent to which the mutant H-Ras activated signaling in the MAPK and PI3K pathway in this model was determined by quantifying the ratio of phosphorylated ERK1/2(Thr202/Try204) to total ERK1/2 and the ratio of pAKT(Ser473) to total AKT. As shown in Supplementary Figure 1B, the basal level of pERK1/2 was significantly increased by 1.6 ± 0.1-fold in H-Ras-expressing cells. Supplementary Figure 1C shows the same type of analysis for pAKT and indicates that there was a 2.7 ± 0.2-fold increase in the H-Ras cells. Thus, in this model the oncogenic H-Ras constitutively activated both the MAPK and PI3K pathways in the 10T1/2 cells.

**Figure 4:**
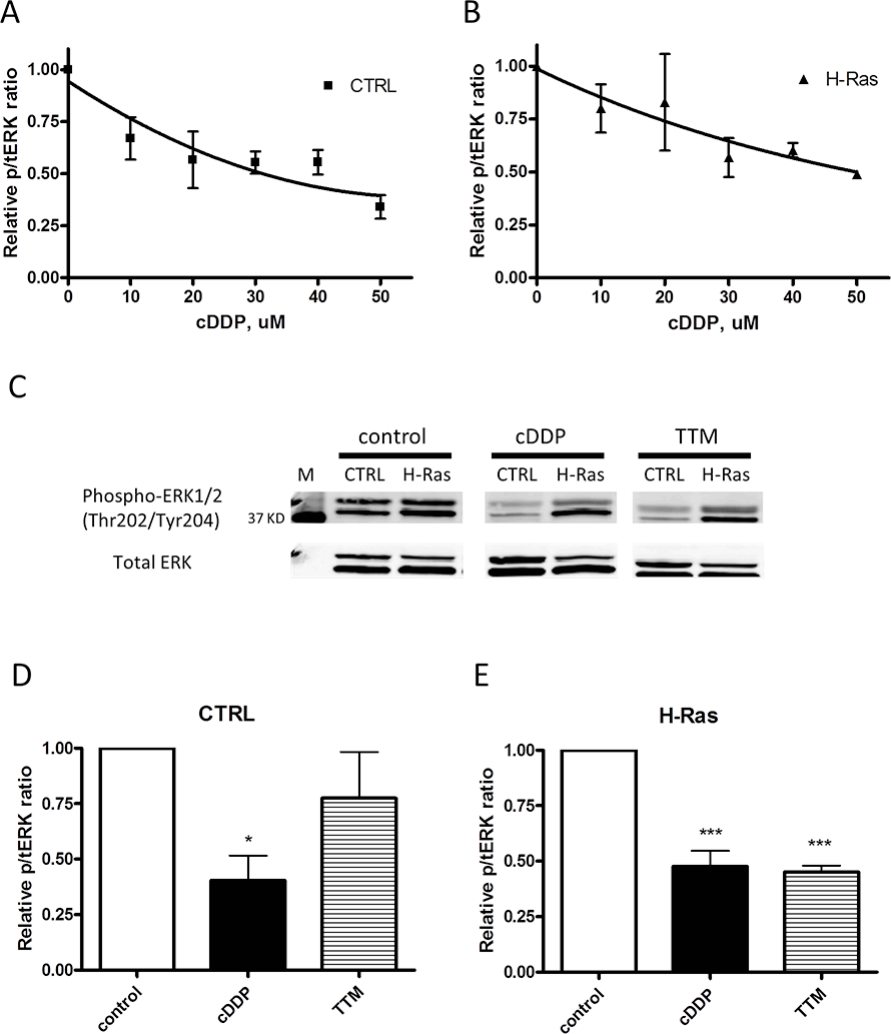
Inhibition of EKR1/2 phosphorylation by cDDP and TTM in whole cells CTRL and H-Ras-expressing cells were treated with increasing concentrations of cDDP for 1 h prior to Western blot analysis. **A.** CTRL cells; **B.** H-Ras transformed cells. **C.** Representative Western blot showing the impact of a 1 h exposure to 30 μM cDDP or TTM on pERK and total ERK in CTRL and H-Ras transformed cells. **D. and E.** Histograms summarizing change in ratio of pERK1/2 to total ERK1/2 in 3 independent Western blots. Data are presented as mean ± SEM. *p < 0.05; *** *p* < 0.001

The effect of cDDP and the Cu chelating agent tetrathiomolybdate (TTM) on the activity of the MAPK pathway was assayed by quantifying changes in the ratio of pERK to total ERK1/2 by Western blot analysis following exposure to increasing concentrations of cDDP for 1 h. Figure 4A and 4B show that cDDP inhibited the phosphorylation of ERK1/2 with an IC_50_ of 36.4 ± 4.9 μM in CTRL cells and an IC_50_ of 35.2 ± 0.3 μM in the H-Ras cells. The potency of cDDP was compared to that of TTM. As shown in Figure 4C–4E, in the CTRL cells pERK was reduced to 40 ± 11% of that in the untreated cells following a 1 h exposure to 30 μM cDDP; in the H-Ras cells the reduction was to 48 ± 7% of control. In contrast, TTM produced a significant reduction only in the H-Ras expressing cell line to 45 ± 3% of control. Thus, while cDDP inhibited the MAPK pathway in both cell types, Cu chelation was effective only when activity in the pathway was augmented by oncogenic H-Ras expression. This effect of TTM is consistent with reports that MEK1/2 is a Cu-dependent enzyme and that oncogenic transformation increases dependency on Cu [13, 15].

### Cu blocks the effect of cDDP on the MAPK pathway

**Figure 5:**
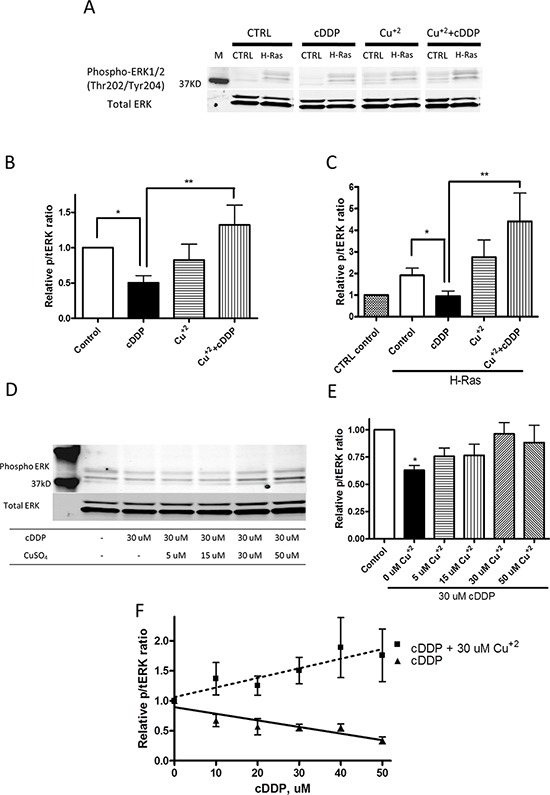
Cu counteracts cDDP-induced inhibition of ERK phosphorylation in whole cells **A.** Representative Western blot showing increased pERK in H-Ras cells. **B.** and **C.** Histograms summarizing change in ratio of pERK1/2 to total ERK1/2 from the analysis of 3 independent Western blots. **D.** Representative Western blot showing effect of increasing concentration of Cu^+2^ on level of pERK1/2. **E.** Histogram summarizing change in ratio of pERK1/2 to total ERK1/2 from the analysis of 3 independent Western blots. **F.** Effect of cDDP alone or in combination with 30 μM Cu^+2^ as a function of cDDP concentration on ratio of pERK1/2 to total ERK1/2. Data are presented as mean ± SEM. *p < 0.05; **p < 0.01.

The ability of cDDP to bind to CXXC motifs in proteins responsible for Cu homeostasis suggested the possibility that cDDP may limit the availability of Cu to MEK by interfering with the loading of Cu onto the enzyme. To address this point, we tested the ability of Cu to offset the cDDP-induced reduction in pERK1/2. The cells were incubated with either 30 uM CuSO_4_ or with 30 uM cDDP alone or in combination with Cu for 1 h. Figure 5A shows a representative Western blot, and Figures 5B and 5C show the values for the level of pERK1/2 determined from at least 3 independent experiments. Exposure to CuSO_4_ alone produced no effect in the CTRL cells and only a small and statistically insignificant increase in pERK1/2 level in the H-Ras cells, whereas cDDP reduced its level in both. CuSO_4_ reversed the cDDP-induced reduction in pERK1/2 in both cell types; in the H-Ras cells it actually increased pERK by 1.6 ± 0.4-fold (*p* = 0.030). Figures 5D and 5E show that the ability of Cu to reverse the effect of cDDP in the CTRL cells was concentration-dependent over the range of 5 – 30 uM CuSO_4_ and that it reached a plateau above 30 uM Cu. The data in Figure 5F show that the ability of 30 μM to reverse the effect of cDDP increased in proportion to the degree of inhibition produced by cDDP and that, under these circumstances, Cu actually stimulated MEK1/2 activity. These results are consistent with the concept that inhibition by cDDP can be reversed by Cu in whole cells, but suggest a complex rather than simple competitive interaction.

In cells expressing mutant Ras genes ERK1/2 is activated by phosphorylation primarily by MEK1/2 which has been identified as a Cu-dependent enzyme [13, 15]. To exclude the possibility that cDDP interferes with the pathway upstream of MEK1/2, we analyzed the effect of a 1 h exposure to 30 uM cDDP on the activation of MEK1/2 as detected by Western blot analysis using an antibody that detects the MEK(Ser217/221) phosphorylation. As shown in Supplementary Figure 2A and 2B, the ratio of pMEK to total MEK was significantly higher in the H-Ras than in the CTRL cells. However, cDDP did not reduce pMEK in either cell type, nor did Cu increase the extent of MEK phosphorylation (Supplementary Figure 2C and 2D). This finding indicates that cDDP does not impair steps in the pathway between H-Ras and MEK, that these upstream steps are not augmented by Cu, and that cDDP reduces pERK through an effect on the activity of MEK.

### cDDP does not acutely reduce intracellular Cu

**Figure 6:**
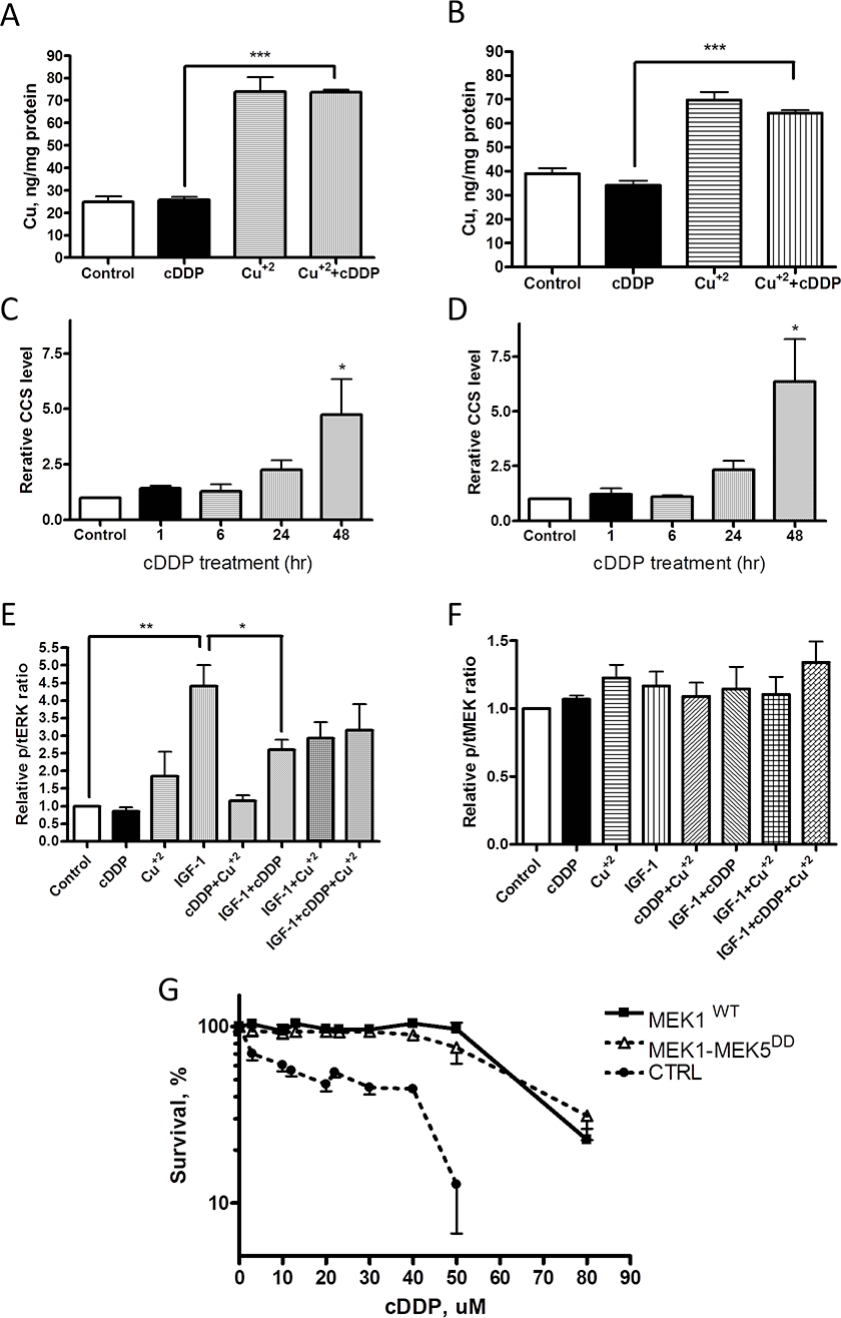
Effect of cDDP on whole cell Cu content, CCS level and IGF-1 signaling and MEK1-MEK5^DD^ activity Cu content was determined by ICP-MS following incubation of CTRL cells **(A)** and H-Ras-expressing cells **(B)** with 30 uM cDDP alone, Cu^+2^ alone or the combination (*N* = 4). Change in CCS protein as a function of time during incubation of cells with 30 μM cDDP in CTRL **(C)** and H-Ras **(D)** cells. **E.** Relative ratio of pERK1/2 to total ERK1/2, and **(F)** of pMEK1/2 to total MEK1 in response to IGF-1 following exposure to cDDP, Cu^+2^ or the combination of both in CTRL cells. **G.** Effect of a 72 h exposure to increasing concentrations of cDDP on MEK1/2 activity in CTRL cells engineered to express wild type MEK1 or MEK1-MEK5^DD^. Data are presented as mean ± SEM, *N* = 3. *, *p* < 0.05; **, *p* < 0.01; ***, *p* < 0.001.

The ability of excess Cu to antagonize cDDP-induced inhibition of MEK1/2 in whole cells raised the question of whether cDDP was working by limiting the access of Cu to the enzyme by reducing the pool of exchangeable Cu in the whole cell thus limiting transfer of Cu to the enzyme. To determine whether cDDP produced an acute reduction in intracellular Cu, the CTRL and H-Ras-expressing cells were exposed to either 30 uM cDDP or Cu alone or in combination for 1 h and whole cell Cu content was determined by ICP-MS. As shown in Figure 6A and 6B, this concentration of cDDP had no effect on the basal level of intracellular Cu, nor did the concurrent addition of cDDP limit the ability of supplementary CuSO_4_ to increase cellular Cu which argues that cDDP did not significantly inhibit Cu influx. However, this leaves open the question of whether cDDP reduces the exchangeable pool of Cu or interferes with the transfer of Cu to MEK1/2. To detect a change in exchangeable Cu we measured the effect of cDDP on the level of the Cu chaperone CCS which is a sensitive measure of the availability of intracellular Cu [16]. cDDP produced a clear time-dependent increase in CCS in 10T1/2 CTRL and H-Ras cells (Figure 6C and 6D); however, this evolved slowly over 48 h whereas the same concentration of cDDP reduced MEK activity within 1 h. Thus, it appears unlikely that cDDP inhibits MEK1/2 by abruptly reducing the availability of Cu.

As an independent test of whether cDDP acutely limits the availability of intracellular Cu we took advantage of a previous observation that deficiency of Cu due to the loss of the influx transporter CTR1 impairs signaling from multiple receptor tyrosine kinases including the insulin/IGF1 receptor [13, 14]. CTRL cells were exposed to10 nM IGF-1 for 60 min following a 1 h exposure to 30 μM cDDP or control medium. As shown in Figure 6E, while cDDP blocked insulin receptor signaling this was only partially reversed by supplementing the cell with 30 μM Cu. IGF-1 did not significantly alter the level of phospho-MEK nor did cDDP reduce it (Figure 6F). These results provide further evidence against the idea that cDDP acutely reduces exchangeable Cu sufficiently to block MEK1/2.

### cDDP inhibits the activity of Cu-independent MEK

If cDDP inhibits MEK1/2 by reducing its access to Cu, or the ability of bound Cu to configure MEK1 in an active form, then a form of MEK that does not require Cu for activity should not be inhibited. The CTRL cells were engineered to express a Cu-independent form of MEK consisting of the ERK1/2 binding region of MEK1 fused to a constitutively active kinase domain of MEK5 (S311D/T315D) to create MEK1-MEK5^DD^ [15]. Figure 6G shows that 10T1/2 cells engineered to express either MEK1^WT^ or MEK1-MEK5^DD^ were more resistant to cDDP than the CTRL cells infected with empty vector. The IC_50_ for the CTRL cells was 21.8 ± 9.6 μM whereas it was 59.5 ± 9.2 μM for the MEK1^WT^ and 68.6 ± 3.9 μM (*p* < 0.01 for both) for the MEK1-MEK5^DD^-expressing cells indicating that high level expression of MEK1 by itself can confer 2.7-fold resistance but that cDDP was equally effective against cells over-expressing either form of the enzyme. This result establishes that cDDP can inhibit the enzyme activity irrespective of whether the activity of the kinase activity is Cu-dependent.

### cDDP and Cu bind to phospho- and total MEK1/2 in whole cells

**Figure 7:**
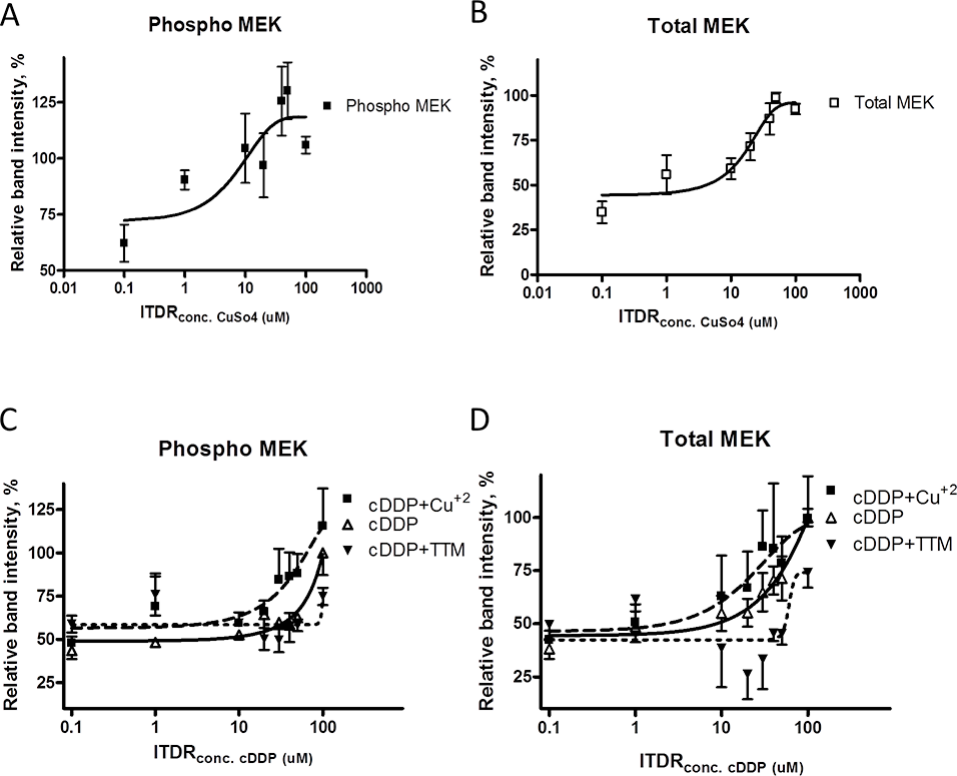
Thermal stabilization of pMEK1/2 and MEK1/2 by cDDP in whole cells and modulation by Cu and TTM ITDR curves for the stabilization of pMEK1/2 **A.** and total MEK1/2 **B.** by cDDP at 45°C. Effect of either 30 μM Cu^+2^ or TTM on the ITDR curves for pMEK1/2 **C.** and total MEK1/2 **D.** Data are presented as mean ± SEM, *N* = 3.

To determine whether cDDP binds to phospho- and total MEK1/2 in whole cells, and how stability is influenced by Cu, we evaluated the stability of MEK1/2 in whole cells using the cellular thermal shift assay (CETSA). The ITDRF curves shown in Figure 7A and 7B indicate that Cu increases the stability of both phospho- and total MEK1/2 but only when the medium was supplemented with sufficient CuSO_4_ to raise the total Cu concentration to well above physiologic levels. cDDP also increased the stability of both forms of MEK1/2, and it was effective at the concentrations from 30 to 100 uM at a temperature of 40°C (Figure 7C and 7D). These results provide evidence that both forms of MEK1/2 bind cDDP and that some binding is detectable at concentrations close to the IC_50_. To determine whether the ability of cDDP to bind to the two forms of MEK1/2 was influenced by the availability of Cu, ITDRF curves were constructed for the effect of increasing concentrations of cDDP in the presence of 30 uM Cu or 30 uM TTM. Rather than antagonizing the ability of cDDP to stabilize MEK1/2, Cu augmented this effect consistent with the earlier observation that Cu was more effective at enhancing MEK1/2 activity when it was being inhibited by cDDP. Addition of Cu shifted the ITDRF to the left, and reduction of intracellular Cu by chelation with TTM shifted it to the right indicating that, in whole cells, the ability of cDDP to bind to either form of MEK1/2 was dependent on the availability of Cu. Thus, both Cu and cDDP interact with phospho- and total MEK1/2 in whole cells in a manner that increases their stability, and the interaction of cDDP with both forms of MEK1/2 is favored rather than antagonized by Cu.

### Identification of cDDP and Cu binding sites in MEK1/2

The interaction of cDDP and Cu with MEK1 was modelled using quantum chemistry techniques and the known crystal structure of the enzyme. The minimum number of predicted Cu-binding sites includes Met94-Cys142, Met143-Cys207 and Met249-Cys341. The computational model consists of a representative binding site composed of the side chains of a methionine and a deprotonated cysteine residue (termed prot). The reaction energy for replacing Cu with cDDP is obtained from:

[Cu(prot)(OH2)2](n−1)++cis−[Pt(NH3)2(OH2)2]2+→[Pt(prot)(NH3)2]++[Cu(OH2)4]n+,n=1 or 2

**Figure 8:**
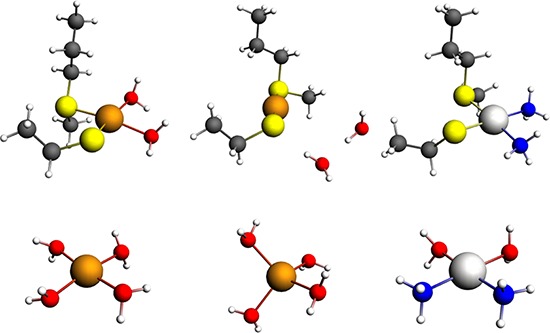
Models of the binding of Cu and Pt to the predicted Met56 and Cys104 site in MEK1/2 Upper row, model of the binding of Cu^+2^ (left), Cu^+1^ (center) and Pt(II) to the Met94-Cys142 site in MEK1. Lower row, model of Cu^+2^ (left), Cu^+1^ (center) and cDDP (right) aqua complexes. Color codes: copper, copper; platinum, platinum; blue, nitrogen; red, oxygen; white, hydrogen.

Calculation of the energies for Cu and Pt (in a cDDP environment) for these sites indicates that Pt has 12–15 kcal/mol lower (more favorable) energy of binding to the site than Cu and thus that Cu would be displaced by Pt (Table 1). The optimized geometries of the models are depicted in Figure 8.

**Table 1:** Reaction energy (kcal/mol) for replacing Cu(I) or Cu(II) with cisplatin in a model binding site composed of Met94-Cys142 Protein backbone atom coordinates were constraint as indicated.

Constraint	Cu(I)	Cu(II)
**Cα**	−12.1	−14.1
**Cα and Cβ**	−15.7	−13.7

## DISCUSSION

The ability of cDDP to bind to ATOX1 and the metal binding domains of ATP7B sparked our interest in whether cDDP could bind to and interfere with the function of other Cu-binding proteins. MEK1/2 has recently been identified as a Cu-binding protein [13] whose activity is compromised when whole cell Cu levels are reduced by chelation [15]. The Cu-dependency of MEK1/2 becomes exposed when the MAPK pathway is driven by activating mutations in BRAF, and under these circumstances treatment with the Cu chelator TTM reduces the growth of human melanoma xenografts [15]. Exactly how Cu is bound in MEK1/2 is not yet clear, but the Cu binding site is quite different from the CXXC motif to which cDDP is known to bind in ATOX1 [6, 8, 17, 18] and ATP7B [9, 10, 19]. Not very much is known about the types of cDDP binding sites in proteins and we hypothesized that the range of motifs to which cDDP can bind may include those found in other Cu binding proteins such as MEK1/2.

The results reported here indicate that cDDP can inhibit MEK1/2 activity at clinically relevant concentrations. Using ERK1 as a substrate, cDDP was found to inhibit the activity of a recombinant form of MEK1 with an IC_50_ of 0.28 μM and that this was antagonized by supplementary Cu in the form of either Cu^+1^ or Cu^+2^. Two types of thermal denaturation assays indicated that, like Cu, cDDP binds to recombinant MEK1. Studies using parental and H-Ras oncogene-transformed mouse fibroblasts demonstrated that cDDP reduced the ratio of pERK1/2 to total ERK1/2 in whole cells, a classic measure of the activity of the MAPK pathway, and that this was antagonized by supplementary Cu indicating that Cu influences cDDP-induced inhibition of MEK1/2 in this setting as well. Curiously, the ability of Cu to antagonize cDDP-induced reduction of pERK was greater at higher levels of inhibition and this resulted in an increase in MEK1/2 activity to above baseline. This is discussed further below. The fact that cDDP had no effect on the ratio of pMEK1/2 to total MEK1/2 suggests that it does not interfere with the activating phosphorylation of this enzyme by upstream kinases.

The data from the studies of recombinant MEK1 indicate that both oxidized and reduced Cu affects the ability of cDDP to inhibit the enzyme. One possibility is that Cu and cDDP compete for the same binding site, but another is that a conformational change produced by the binding of cDDP may modulate the loading of Cu onto a separate binding site and *vice versa*. Recent studies have documented that cDDP and Cu can simultaneously bind to cysteines [18] and that the binding of Cu facilitates the binding of cDDP and limits its departure to other thiols such as GSH [20, 21]. Molecular modeling and quantum chemistry calculations identified 3 predicted Cu and cDDP binding sites in MEK1 and indicated that cDDP would be expected to readily displace Cu from all of these sites.

In whole cells the situation is more complex. Supplementary Cu^+2^ that is subsequently reduced to Cu^+1^ before cellular entry enhanced and cDDP inhibited MEK1/2 activity but the fact that cDDP inhibited the activity of MEK1-MEK5^DD^ as well as wild type MEK1/2 indicates that its ability to inhibit does not depend on Cu^+1^ activation of the kinase. In addition to this type of interaction at the enzyme itself, in whole cells there is also the possibility that cDDP interferes with the transfer of Cu^+1^ to MEK1/2. It is not currently known how MEK1/2 acquires Cu, but two lines of evidence argue against the possibility that cDDP inhibits MEK1/2 by reducing Cu^+1^ availability in the whole cell. First, at concentrations that inhibited MEK1/2 activity cDDP did not produce an acute change in whole cell Cu. Second, while cDDP reduced IGFR1 signaling this was not clearly offset by supplementary Cu^+2^ suggesting it was not due to reduced Cu availability. It is important to note that these are both quite insensitive measures of the availability of intracellular Cu, and that recent studies using fluorescent sensors detect an exchangeable pool that could potentially be modulated by cDDP in the absence of a change in whole cell Cu content [22–25]. Separate from whether cDDP reduces the concentration of available Cu, cDDP may interfere with the transfer of Cu^+1^ from the one or more unknown donors that provide Cu^+1^ to MEK1/2. Like Cu, ATOX1 has now been shown to transfer cDDP to both the second and fourth metal binding domains of ATP7B [8, 10]. Since cDDP binds to the same CXXC motif as Cu this suggests that cDDP has the potential to interfere with Cu transfer reactions involving these and potentially other Cu binding motifs.

The thermal denaturation studies indicated that both Cu and cDDP bind to recombinant MEK1 and that both stabilize MEK1/2 in whole cells. In each setting the binding was influenced by the availability of Cu. Additional Cu^+2^ enhanced rather than hindered the ability of cDDP to protect MEK1/2 from thermal denaturation in whole cells whereas reducing the availability of intracellular Cu by chelating it with TTM reduced the ability of cDDP to stabilize MEK1/2, thus documenting that this was a direct effect of Cu. This suggests a model in which the Cu and cDDP bind to different sites, and that the conformational shift that probably occurs when MEK1/2 binds Cu actually assists cDDP to stabilize the protein. Stated another way, the conformational shift produced when both metals are bound may result in greater stability than can be achieved by either alone. There is already some evidence that Cu and cDDP can co-occupy the same CXXC motif in the Cu chaperone ATOX1 [8] which hints at the potential of these metals to interact in a complex manner at other types of Cu binding sites. This would provide an explanation for the observation that, while cDDP alone inhibits MEK1/2 activity in whole cells, the addition of Cu^+2^ not only restores activity it actually increases it substantially. We speculate that the sum of the changes induced by each metal results in a conformation whose activity or ability to bind substrate is even greater than that of the protein when loaded only with Cu. While it is pMEK1/2 that has the bulk of the enzymatic activity, it is noteworthy the cDDP stabilized both pMEK and total MEK to the same degree. Whatever change cDDP produces in MEK1/2 it does not perturb the relative amount of pMEK indicating that the cDDP does not limit the activating phosphorylation which in turn suggests that it does not hinder the ability of upstream kinases to access the MEK1/2.

The discovery that cDDP inhibits MEK1/2 in whole cells at clinically relevant concentrations begs the question of whether this effect contributes importantly to the cytotoxic potential of cDDP or whether the extensive DNA damage done by this drug dwarfs any pro-apoptotic contribution made by impairment of signaling in the MAPK pathway. Synergy between small molecule inhibitors of the MAPK pathway and cDDP has been reported in non-clinical studies [26, 27] pointing to the possible enhanced efficacy of cDDP and MEK1/2 inhibitors in patients. However, while such synergy could reflect independent inhibition of MEK1/2 by each drug independently, it could also result from inability of the inhibited MAPK pathway to fully participate in the anti-apoptotic cellular injury response triggered by DNA damage. In any case, it is noteworthy that cDDP was effective at inhibiting MEK1/2 both in the parental cells and when the MAPK pathway was being driven hard by an oncogenic H-Ras.

## MATERIALS AND METHODS

### Drug and reagents

All chemicals and solvents were purchased from Cell Signaling Technologies (Danvers, MA). cDDP was purchased from Teva Parenteral Medicines, Inc (Irvine, CA). Ammonium tetrathiomolybdate (TTM) was purchased from Sigma-Aldrich (St. Louis, MO).

### Cell culture

The wild type 10T1/2 cells (CTRL) and sublines transformed with H-Ras oncogenes were kindly provided by Dr. Peter Vogt (Scripps Research Institute, La Jolla, CA). Cells were cultured DMEM high-glucose media (Thermo scientific, Waltham, MA) supplemented with 10% FBS, 100 U/mL penicillin, and 100 μg/mL streptomycin (Mediatech, Inc. Manassas, VA) in 5% CO2 in air at 37°C. In experiments where medium was supplemented with Cu the source of the Cu was CuSO_4_ either alone, identified as Cu^+2^, or in combination with 5-fold excess of DTT relative to the protein concentration, identified as Cu^+1^.

### Soft-agar colony formation assay

10T1/2 CTRL and H-Ras cells were removed from culture plates with trypsin and were plated at 2 × 10^3^ cells in 0.3% low melting point agarose/growth medium onto six-well plates with a 0.6% agarose underlay. About 1.0 ml growth medium was added on the top of agarose and the medium was changed every 3–4 days, and incubated in 5% CO_2_ in air at 37°C for 14 days. At the end of incubation, the cells were washed with PBS and incubated in a solution of 0.005% crystal violet for 60 min and then rinsed with water. Then, the number of colonies were counted.

### CCK-8 assay

10T1/2 CTRL and H-Ras cells (1×10^3^ cells/well in 96-well culture plates) were treated with 0.1% DMSO or different concentration of cDDP (3, 10, 15, 20, 30 and 50 uM) for 72 h after which 5 ul of CCK-8 solution was added to each well and incubated for 4 h at 37°C. Absorbance was measured at 450 nm on a microplate reader (VERSAmax, Moleuclar Devices, Sunnyvale, CA). The cell viability (%) was calculated relative to the control from the mean of pooled data from 3 independent experiments with 6 wells for each drug concentration.

### Western blotting

Cells were dissolved RIPA lysis buffer containing protease and phosphatase inhibitor cocktail (Thermo scientific, Lorgan, UT) for 30 min. The lysates were centrifuged at 14100g for 10 minutes at 4°C. The resulting supernatant was subjected to protein content determination using a detergent-compatible protein assay kit, DCTM Protein Assay (Bio-Rad, Hercules, CA). Thirty microgram of total protein were loaded onto 4 to 15% SDS-polyacrylamide gels and electrotransferred to Immobilon PVDF membrane (Millipore, Billerica, MA). Blots were incubated over night at 4°C in primary antibody at 1:1000 dilution. These were then rinsed 3 times in TBS-T (0.1% Tween) and incubated with the appropriate Li-Cor secondary antibodies conjugated with 680LT or 800CW infrared dyes (Li-Cor Biosciences, Lincoln, NE). Sources of antibodies were as follows: monoclonal antibodies against total p44/42 MAPK (ERK1/2), MEK1 (1:2000 dilution), polyclonal antibodies against phospho-p44/42 (pERK1/2), (Thr202/Tyr204), phospho-MEK1/2 (Ser217/221), anti-alpha-tubulin (Cell Signaling, Boston, MA), anti-CCS (Santa Cruz Biotechnology, Dallas, TX). The blots were visualized and quantified using a Li-Cor Odyssey Imager (Li-Cor Bioscience, Lincoln, NE).

### Intracellular Cu measurement

For measurement of intracellular ^63^Cu accumulation, 10T1/2tva cells were plated at 7.5 × 10^5^ cells/well into 6 well plates in DMEM containing 10% fetal bovine serum. An extra plate of 6 cultures was prepared for measurement of protein concentration. 10T1/2 CTRL and H-Ras cells were treated with 30 μM cDDP or TTM for 1 h and then washed 3 times with ice-cold PBS. The protein content of replicate cultures was measured using the detergent-compatible DCTM Protein Assay (Bio-Rad, Hercules, CA). Treated cells were lysed with 214 μl of 70% nitric acid and incubated at room temperature overnight followed by heating to 65°C for an additional 16 h. The resulting cell lysates were diluted with 6 ml of buffer for ICP-MS containing 0.1% Triton-X, 1% nitric acid and 1 ppb indium. Cu concentrations were measured using inductively coupled plasma mass spectrometry (ICP-MS). The Cu content was normalized to protein content.

### Cellular thermal shift assay (CETSA)

The ability of cDDP to bind to and stabilize MEK against thermal denaturation in whole cells, and as a recombinant protein, was tested using the cellular thermal shift assay as described previously [28]. In brief, 10T1/2 CTRL cells were seeded in 15cm cell culture plates and exposed to 30 uM cDDP. After a 1 h exposure, the cells were washed with PBS and suspended in PBS containing complete protease inhibitor cocktail. These aliquots were heated individually at different temperatures for 3 min followed by cooling at room temperature for 3 min. The cells were then dissolved using RIPA buffer and the lysates were analyzed by Western blotting. Alpha-tubulin levels were used to normalize the band intensity. The interaction of cDDP with human recombinant MEK1 (Enzo Life Science, Farmingdale, NY) was also tested by CETSA. Fifty ng of recombinant MEK1 was treated with 30 uM cDDP for 30 min at 30°C and aliquots were then heated individually at various temperatures for 3 min followed by cooling at room temperature for 3 min and analyzed by Western blotting. For both types of CETSA the band intensities in the heated samples were related to the intensity of the lowest temperature.

To evaluate the drug concentration effect, isothermal dose-response procedure was performed. 10T1/2 CTRL cells were treated with variety concentration of cDDP for 1 hr. Then cells were heated at 40°C for 3 min followed by 3 min cooling at room temperature. The levels of phospho- and total ERK or MEK in cell lysates were measured by western blotting. For the recombinant protein, 50 ng of recombinant MEK were treated with variety concentration of cDDP or Cu at 30°C for 30 min. Then these were heated at 45°C for 3min followed by 3 min cooling at room temperature. The existences of MEK were measured by western blotting. The band intensities were related to the sample treated with 100 uM cDDP as a control. And these data were fitted using a nonlinear regression curve fit (Bolzmann sigmoidal).

### Differential scanning fluorimetry (DSF)

SYPRO orange dye 5000x (Sigma-Aldrich, St. Louis, MO, USA) was dissolved in DMSO at 10% (v/v) and kept at −80°C. Prior to use, the dye stock was diluted 1:4 in water. Recombinant MEK1 protein was diluted in MES (2-(N-morpholino)ethanesulfonic acid) buffer (10 mM, pH 7.0) and 100x SYPRO orange dye was added as 10% (v/v) such that the final concentration of protein was 2 uM. Twenty five uL of reaction mixture was transferred to Fast Optical 96 Well Reaction Plate (Applied Biosystems, Foster City, CA) and kept on ice. The plate was sealed with Optical Adhesive Film (Applied Biosystems, Foster City, CA) and centrifuged at 500 g for 2 min to remove air bubbles. Fluorescence intensity was measured over the temperature range starting from 10 to 90°C (0.02°C/sec) using a ViiA™ 7 Real-Time PCR System (Applied Biosystems, Foster City, CA) with an appropriate filter. The melting temperature (Tm) was calculated on normalized data using a Boltzmann sigmoid equation as previously described. [29]

### MEK activity assay

The effect of cDDP on the activity of activated recombinant MEK1 was evaluated using MAPK1 (ERK2) (Life technologies, Carlsbad, CA) as a substrate. Human recombinant MEK1 was exposed to increasing concentrations of cDDP or the MEK inhibitor U0126 (Cell Signaling, Boston, MA) for 30 min at 30°C following which human recombinant MAPK1 (ERK2) was added for an additional 30 min at 30°C. The levels of total and phospho-ERK were measured by Western blotting. Relative band intensities of phospho and total ERK were used as a measure of MEK activity, and change in the ratio of these band intensities was taken as an indication of altered MEK activity. In all cases data on band intensities were normalized to the ratio in the CTRL cells.

### Plasmids and plasmid engineering

pBABEpuro-HA-MEK1-MEK5^DD^ and pWZLblasti-HA-MEK1-MEK5^DD^ (S311D/T315D; DD) were kind gift from Drs. Donita Brady and Christopher Counter (Department of Pharmacology and Cancer Biology, Duke University Medical Center, Durham, NC). pWZLblasti-HA-MEK1^WT^ was obtained from Addgene (Cambridge, MA). 10T1/2 CTRL cells were infected with MuLV retrovirus pUMVC3 (Addgene, Cambridge, MA) prepared from these vectors according to the manufacturer's protocol. Expression of wild type and MEK1-MEK5^DD^ was confirmed by Western blot analysis using an antibody to the HA tag.

### Quantum chemistry calculations

Our calculations are based on the crystal structure of MEK1 (pdb ID 3eqd). The positions of either the Cα or the Cα and Cβ atoms of the protein side chains were constrained to their positions in the crystal structure to mimic a protein environment that is flexible or rigid. All calculations were performed with the ADF software (http://www.scm.com) [30] package using density functional theory with the B3LYP exchange-correlation functional and a triple-zeta basis set of Slater type functions with polarization functions and frozen core approximation (TZP). Scalar relativistic effects were included by means of the zeroth-order regular approximation (ZORA). Environmental effects were included as a dielectric continuum with the COSMO model using a dielectric constant of 18.5 which corresponds the interfacial region between protein and aqueous environment.

### Statistical analysis

All experiments were repeated at least 3 times. All values are reported as mean ± SEM. The Student *t* test was use to compare mean values with assumption of unequal variance; *p* < 0.05 was considered significant. The statistical analyses were performed using the JMP ver. 5.0.1 software package (SAS institute Inc, Cary, NC). For ITDR CETSA curve, the data were analyzed using GraphPad Prism 4 software (GraphPad Software Inc., La Jolla, CA).

## SUPPLEMENTARY FIGURES



## References

[1] Casagrande N, De Paoli M, Celegato M, Borghese C, Mongiat M, Colombatti A, Aldinucci D (2013). Preclinical evaluation of a new liposomal formulation of cisplatin, lipoplatin, to treat cisplatin-resistant cervical cancer. Gynecol Oncol.

[2] Abada P, Howell SB (2010). Regulation of cisplatin cytotoxicity by Cu influx transporters. Met Based Drugs.

[3] Rae TD, Schmidt PJ, Pufahl RA, Culotta VC, O'Halloran TV (1999). Undetectable intracellular free copper: the requirement of a copper chaperone for superoxide dismutase. Science.

[4] Banci L, Bertini I, Ciofi-Baffoni S, Kozyreva T, Zovo K, Palumaa P (2010). Affinity gradients drive copper to cellular destinations. Nature.

[5] Howell SB, Safaei R, Larson CA, Sailor MJ (2010). Copper transporters and the cellular pharmacology of the platinum-containing cancer drugs. Mol Pharmacol.

[6] Boal AK, Rosenzweig AC (2009). Crystal structures of cisplatin bound to a human copper chaperone. J Am Chem Soc.

[7] Arnesano F, Banci L, Bertini I, Felli IC, Losacco M, Natile G (2011). Probing the interaction of Cisplatin with the human copper chaperone atox1 by solution and in-cell NMR spectroscopy. J Am Chem Soc.

[8] Palm-Espling ME, Andersson CD, Bjorn E, Linusson A, Wittung-Stafshede P (2013). Determinants for Simultaneous Binding of Copper and Platinum to Human Chaperone Atox1: Hitchhiking not Hijacking. PLoS One.

[9] Safaei R, Adams PL, Mathews RA, Manorek G, Howell SB (2013). The role of metal binding and phosphorylation domains in the regulation of cisplatin-induced trafficking of ATP7B. Metallomics.

[10] Dolgova NV, Nokhrin S, Yu CH, George GN, Dmitriev OY (2013). Copper chaperone Atox1 interacts with the metal-binding domain of Wilson disease protein in cisplatin detoxification. Biochem J.

[11] van Boom S, Chen BW, Teuben JM, Reedijk J (1999). Platinum-Thioether bonds can be reverted by guanine-N7 bonds in Pt(dien)2+ model adducts. American Chemical Society.

[12] Haremaki T, Fraser ST, Kuo YM, Baron MH, Weinstein DC (2007). Vertebrate Ctr1 coordinates morphogenesis and progenitor cell fate and regulates embryonic stem cell differentiation. Proc Natl Acad Sci U S A.

[13] Turski ML, Brady DC, Kim HJ, Kim BE, Nose Y, Counter CM, Winge DR, Thiele DJ (2012). A novel role for copper in Ras/mitogen-activated protein kinase signaling. Mol Cell Biol.

[14] Tsai CY, Finley JC, Ali SS, Patel HH, Howell SB (2012). Copper influx transporter 1 is required for FGF, PDGF and EGF-induced MAPK signaling. Biochem Pharmacol.

[15] Brady DC, Crowe MS, Turski ML, Hobbs GA, Yao X, Chaikuad A, Knapp S, Xiao K, Campbell SL, Thiele DJ, Counter CM (2014). Copper is required for oncogenic BRAF signalling and tumorigenesis. Nature.

[16] Bertinato J, Iskandar M, L'Abbe MR (2003). Copper deficiency induces the upregulation of the copper chaperone for Cu/Zn superoxide dismutase in weanling male rats. J Nutr.

[17] Palm ME, Weise CF, Lundin C, Wingsle G, Nygren Y, Bjorn E, Naredi P, Wolf-Watz M, Wittung-Stafshede P (2011). Cisplatin binds human copper chaperone Atox1 and promotes unfolding *in vitro*. Proc Natl Acad Sci U S A.

[18] Palm-Espling ME, Lundin C, Bjorn E, Naredi P, Wittung-Stafshede P (2014). Interaction between the Anticancer Drug Cisplatin and the Copper Chaperone Atox1 in Human Melanoma Cells. Protein and peptide letters.

[19] Safaei R, Adams PL, Maktabi MH, Mathews RA, Howell SB (2012). The CXXC motifs in the metal binding domains are required for ATP7B to mediate resistance to cisplatin. J Inorg Biochem.

[20] Xi Z, Guo W, Tian C, Wang F, Liu Y (2013). Copper binding promotes the interaction of cisplatin with human copper chaperone Atox1. Chem Commun (Camb).

[21] Xi Z, Guo W, Tian C, Wang F, Liu Y (2014). Copper binding modulates the platination of human copper chaperone Atox1 by antitumor trans-platinum complexes. Metallomics.

[22] Domaille DW, Zeng L, Chang CJ (2010). Visualizing Ascorbate-Triggered Release of Labile Copper within Living Cells using a Ratiometric Fluorescent Sensor. J Am Chem Soc.

[23] Dodani SC, Domaille DW, Nam CI, Miller EW, Finney LA, Vogt S, Chang CJ (2011). Calcium-dependent copper redistributions in neuronal cells revealed by a fluorescent copper sensor and X-ray fluorescence microscopy. Proc Natl Acad Sci U S A.

[24] Dodani SC, Leary SC, Cobine PA, Winge DR, Chang CJ (2011). A targetable fluorescent sensor reveals that copper-deficient SCO1 and SCO2 patient cells prioritize mitochondrial copper homeostasis. J Am Chem Soc.

[25] Dodani SC, Firl A, Chan J, Nam CI, Aron AT, Onak CS, Ramos-Torres KM, Paek J, Webster CM, Feller MB, Chang CJ (2014). Copper is an endogenous modulator of neural circuit spontaneous activity. Proc Natl Acad Sci U S A.

[26] Fu X, Feng J, Zeng D, Ding Y, Yu C, Yang B (2014). PAK4 confers cisplatin resistance in gastric cancer cells via PI3K/Akt- and MEK/Erk-dependent pathways. Biosci Rep.

[27] Huang YS, Xue Z, Zhang H (2015). Sorafenib reverses resistance of gastric cancer to treatment by cisplatin through down-regulating MDR1 expression. Med Oncol.

[28] Molina DM, Jafari R, Ignatushchenko M, Seki T, Larsson EA, Dan C, Sreekumar L, Cao Y, Nordlund P (2013). Monitoring drug target engagement in cells and tissues using the cellular thermal shift assay. Science.

[29] Niesen FH, Berglund H, Vedadi M (2007). The use of differential scanning fluorimetry to detect ligand interactions that promote protein stability. Nat Protoc.

[30] te Velde G, Bickelhaupt FM, Baerends EJ, Fonseca Guerra C, van Gisbergen SJA, Snijders JG, Ziegler T (2001). Chemistry with ADF. J Comput Chem.

