# Supplementary material for: Cisplatin inhibits MEK1/2
Source: Oncotarget. 2015 Jun 20;6(27):23510–22. doi: 10.18632/oncotarget.4355 (PMC4695133; doi:10.18632/oncotarget.4355)
Supplement: Supplementary file 1 [file oncotarget-06-23510-s001.pdf]

SUPPLEMENTARY FIGURES

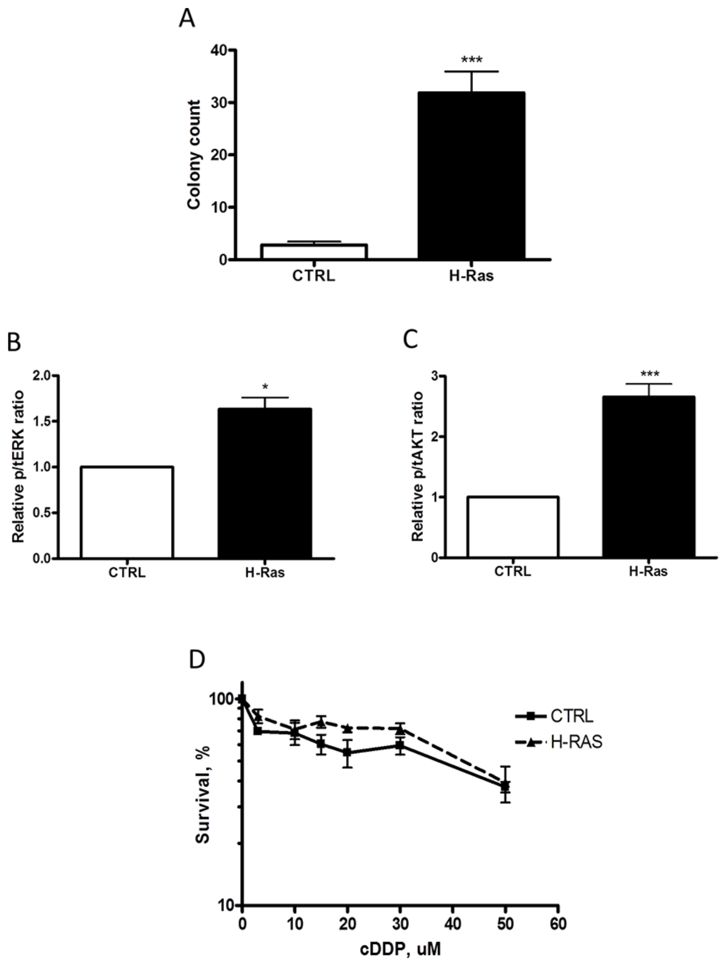

Supplementary Figure 1: Characteristics of CTRL and H-Ras transformed 10T1/2 cells. A. ability to form colonies in soft agar. **B.** relative change in the ratio of phosphoERK1/2 (pERK) to total ERK1/2 (tERK). **C.** relative change in the ratio of phospho AKT to total AKT. **D.** effect of a 1 h exposure of cDDP to increasing concentration of cDDP on CTRL and H-Ras cells. Data are presented as mean ± SEM, $N = 4$.

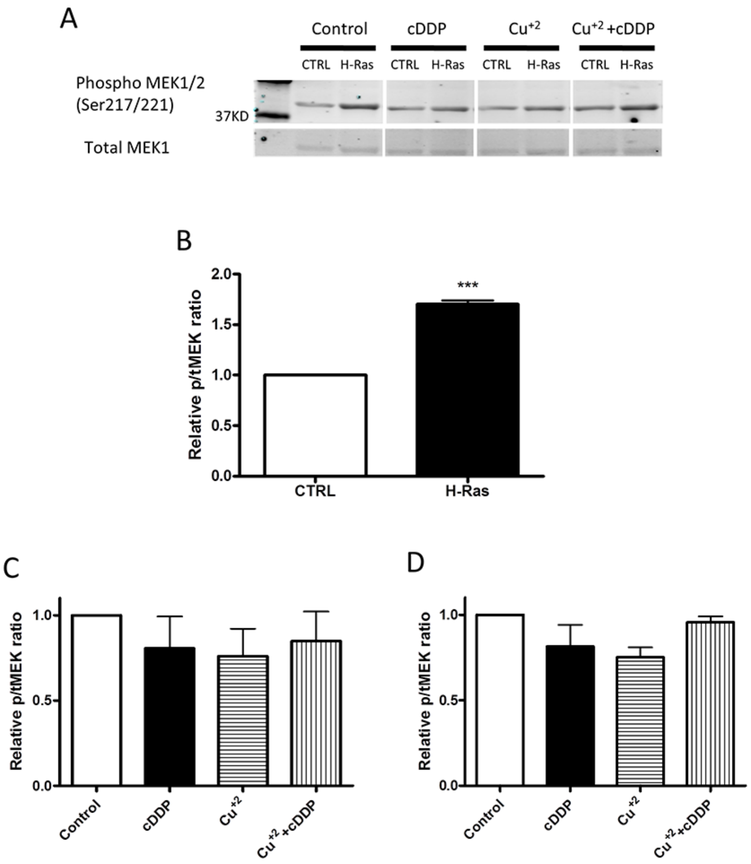

Supplementary Figure 2: Effect of cDDP on the ratio of pMEK1/2 to total MEK1/2 in CTRL and H-Ras-expressing cells. A. and **B.** representative Western blot and histogram showing data from analysis of 3 independent Western blots. **C.** and **D.** histogram showing change in ratio of pMEK to total MEK following 1 h exposure to 30 µM cDDP or Cu^{+2} alone or in combination in the CTRL and H-Ras-expressing cells; $N = 3$. Vertical bars, ± SEM. $*p < 0.05$.
